# Adherence to International Guidelines for the Treatment of Uncomplicated Urinary Tract Infections in Lebanon

**DOI:** 10.1155/2018/7404095

**Published:** 2018-02-20

**Authors:** Wissam K. Kabbara, Mohamad M. Meski, Wijdan H. Ramadan, Dina S. Maaliki, Pascale Salameh

**Affiliations:** ^1^Department of Pharmacy Practice, School of Pharmacy, Lebanese American University (LAU), Byblos, Lebanon; ^2^School of Pharmacy, Lebanese American University (LAU), Byblos, Lebanon; ^3^Faculty of Medical Sciences, Lebanese University, Hadath, Lebanon

## Abstract

**Objective:**

The purpose of this study is to evaluate antibiotic-prescribing practices and adherence to IDSA guidelines for the treatment of uncomplicated urinary tract infections in Lebanon.

**Methods:**

This observational prospective study was conducted in 15 community pharmacies in Lebanon over 1 year in adult females. A regimen of nitrofurantoin 100 mg bid for 5 days or fosfomycin 3 grams single dose were considered appropriate. For the bivariate analysis, the chi-square test was used.

**Results:**

A total of 376 patients were included in this study. The prescribed antibiotic was appropriate in 35 percent of the patients. Age (more than 50 years) did not significantly affect the appropriateness of the prescribed antibiotic (*p*=0.508). The frequency of attacks per year (more than 3) negatively affected the choice of antibiotic (*p*=0.025). The dose and duration of the prescribed antibiotic was appropriate in 73 and 58 percent of the patients, respectively, with a significant inappropriate dose and duration with fluoroquinolones as compared to nitrofurantoin and fosfomycin (*p* < 0.001 for the dose and *p*=0.014 for the duration of therapy).

**Conclusions:**

In an era of increasing bacterial resistance, interventions that improve physicians' prescribing practices for uncomplicated urinary tract infections are needed.

## 1. Introduction

Acute bacterial urinary tract infection (UTI) is one of the most prevalent infections encountered in the outpatient setting [1]. UTIs are very common indications for prescription of antibiotics for otherwise healthy women [2]. In the United States alone, the average of patient visits to health-care providers for uncomplicated UTI was 7 million visits per year between 1996 and 2001 [3]. This high prevalence, coupled with a low risk of progression to severe illness associated with UTIs, merits that more emphasis be geared toward the collateral damage linked with the use of antibiotics for this indication in the community setting [4]. The most recent guidelines on treatment of uncomplicated UTI, published in 2010 by the Infectious Diseases Society of America (IDSA) and the European Society for Microbiology and Infectious Diseases (ESCMID), highlighted the importance of this collateral damage while simultaneously drawing attention to the quintessential role played by local susceptibility data [1].

According to IDSA, first-line therapy for the treatment of uncomplicated UTI consists of trimethoprim-sulfamethoxazole (TMP-SMX) 160 mg/800 mg orally twice daily for 3 days in areas in which the resistance rate of *Escherichia coli* to TMP-SMX does not exceed 20%, nitrofurantoin 100 mg orally twice daily for 5 days, fosfomycin 3 grams single oral dose, or pivmecillinam 400 mg orally twice daily for 5 days [1]. Fluoroquinolones and *β*-lactams are recommended as alternative treatments to be prescribed when first-line agents cannot be used [1]. Fluoroquinolones have excellent concentration in the urinary tract, but they have a high propensity for collateral damage and therefore should be reserved for complicated cystitis and pyelonephritis. *β*-Lactams may have lower efficacy than other available agents and require close follow-up for treatment success [1].

Despite clear recommendations for first-line therapy, the adherence to the guidelines by physicians remains low [4–6]. Taur et al. showed a significant increase in the use of ciprofloxacin for the treatment of uncomplicated UTI in the United States, despite the original release of the IDSA guidelines in 1999, while at the same time showing no significant change in the use of TMP-SMX [6]. The use of fluoroquinolones has been associated with infection with methicillin-resistant *Staphylococcus aureus* and with increase in fluoroquinolone resistance in Gram-negative bacilli [7]. The use of fluoroquinolones and broad-spectrum cephalosporins has been repeatedly associated with increased selection of drug-resistant organisms, as well as infection or colonization with multidrug-resistant strains [1].

Nonadherence to guidelines and the increased use of fluoroquinolones have raised concerns regarding antibiotic resistance. In Lebanon, the resistance of *Escherichia coli* and *Klebsiella* spp. to fluoroquinolones has been consistently increasing [8–10]. In 2015, more than half of the strains were resistant to ciprofloxacin [10]. Few studies have been conducted to evaluate antibiotic prescription patterns in Lebanese hospitals and community pharmacies; however, these studies included prescribed antibiotics for any indication [11–14]. The purpose of this study is to evaluate antibiotic-prescribing practices and adherence to IDSA guidelines for the treatment of uncomplicated UTI in Lebanon.

## 2. Methods

### 2.1. Setting and Design

A prospective-observational study was conducted in fifteen community pharmacies in Lebanon, over a period of 1 year from October 2015 to October 2016. Investigators interviewed patients diagnosed with a UTI and visiting community pharmacies to purchase antibiotic treatment. Investigators had a pre-prepared questionnaire while conducting the survey.

### 2.2. Inclusion/Exclusion Criteria

The study included female patients presenting to a community pharmacy diagnosed with uncomplicated UTI, aged 18 years or older, and prescribed antibiotics. Males, patients diagnosed with complicated UTI, and pregnant women were excluded from the study. Generally, UTI in males is considered complicated because the possibility that the infection has ascended to the kidneys/prostate must be considered. Patients diagnosed with chronic kidney disease (CKD), structural renal disease, or a sexually transmitted disease (STD) were also excluded.

### 2.3. Sources of Data

Investigators interviewed patients in community pharmacies in Lebanon and filled out a questionnaire used for data collection. It included questions about patients' demographics, medical history, and pharmacological treatments including drug, dose, and duration of treatment. Information on nonpharmacological treatment and whether or not a culture was obtained was also collected.

The drug choice, dose, and duration of treatment were marked as appropriate or inappropriate; if all three were appropriate, then the overall treatment regimen was considered appropriate. Appropriateness was determined according to IDSA 2010 recommendations; a regimen of nitrofurantoin 100 mg bid for 5 days or fosfomycin 3 grams single dose was considered appropriate [1]. Pivmecillinam is not available in Lebanon and therefore was not considered as a potential regimen. Susceptibility to TMP-SMX for *Escherichia coli* (*E. coli*) in Lebanon is around 50%, so an empiric regimen of TMP-SMX was considered inappropriate, unless a culture was obtained that showed that the pathogen was susceptible to TMP-SMX [8]. A dose of 160/800 mg of TMP-SMX for a duration of 3 days was considered appropriate as per IDSA guidelines [1]. An empiric regimen of a fluoroquinolone was considered inappropriate. Dose appropriateness for fluoroquinolones was based on the package insert of the prescribed drug and a 3-days duration of treatment was considerate appropriate [1]. Drug choice was considered inappropriate for all other antibiotics prescribed; dose and duration appropriateness were assessed according to the leaflet information.

### 2.4. Statistical Analysis

Data were entered and analyzed using SPSS, version 23 (BM SPSS Statistics for Windows, IBM Corp., Armonk, NY). A descriptive analysis was carried out using frequency and percentage for nominal and dichotomous variables, and mean and standard deviation for continuous variables. For the bivariate analysis, the chi-square test was used to compare nominal variables between groups. In all cases, a *p* value < 0.05 was considered statistically significant.

## 3. Results

### 3.1. Sample Description

A total of 376 patients were included in this study; Table 1 summarizes the demographic information and the prescribed antibiotics. More than half of the patients (52%) reported only one UTI attack per year, and the mean age was 38 years. 35 patients (9%) reported antibiotic allergy, most commonly associated with penicillin. Most patients (73%) received nonpharmacological treatment, and a urine culture was obtained in 26% of the patients.

### 3.2. Prescribed Antibiotic

In the 376 patients included, nitrofurantoin was the most frequently prescribed antibiotic (*n*=98, 26%), followed by ciprofloxacin (*n*=71, 19%). A total of 146 (39%) patients were prescribed a fluoroquinolone, 19 patients (5%) were prescribed fosfomycin, and 113 (30%) patients were prescribed other medications including amoxicillin/clavulanic acid, cephalosporins, and macrolides.

### 3.3. Appropriateness: Choice of Antibiotic

One hundred and thirty-one (35%) patients were prescribed an appropriate medication, and 245 (65%) were prescribed an inappropriate medication (Table 2). Age did not significantly affect the appropriateness of the prescribed antibiotic: 36% of patients under the age of 50 were prescribed appropriate medication versus 31.1% of patients over the age of 50 (*p*=0.508) (Table 3). The frequency of UTI attacks per year negatively affected the choice of the antibiotic: 23% of patients with 3 attacks or more per year received an appropriate medication versus 37.5% of patients with less than 3 attacks per year (*p*=0.025) (Table 3).

### 3.4. Appropriateness: Dose

Two hundred and seventy-six (73%) patients were prescribed the right dose, whereas 100 (27%) patients were prescribed an inappropriate dose (Table 2). Neither age nor frequency of UTI attacks per year affected the appropriateness of the dose prescribed (Table 3). Inappropriate doses were most frequent with fluoroquinolones prescriptions. Only 55% of the prescribed doses of fluoroquinolones were appropriate versus 78% of nitrofurantoin prescriptions, 100% of fosfomycin prescriptions, and 90% of prescriptions for other medications (*p* < 0.001) (Table 3).

### 3.5. Appropriateness: Duration

The duration of treatment was appropriate for 219 (58%) patients and inappropriate for 157 (42%) patients. Appropriateness of the duration of treatment was not affected by age nor by the frequency of UTI attacks per year (Table 3). Appropriateness of the duration of treatment was significantly affected by the drug choice: 62% of nitrofurantoin and fosfomycin prescriptions had an appropriate duration of therapy, compared to 49% of the fluoroquinolones prescriptions (*p*=0.014) (Table 3).

### 3.6. Overall Appropriateness: Regimen (Composite of Drug, Dose, and Duration)

Of the 376 patients, 80 (21%) were prescribed an overall appropriate regimen (defined as an appropriate drug, dose, and duration of therapy) and 296 (79%) were prescribed an inappropriate regimen. Age did not affect the overall appropriateness of the regimen prescribed. Patients with 3 UTI attacks or more per year were less likely to receive an appropriate regimen (12%) when compared to patients with a history of less than 3 attacks per year (24%) (*p*=0.30) (Table 3).

## 4. Discussion

The Infectious Diseases Society of America updated their 1999 guidelines for the treatment of women with uncomplicated cystitis and pyelonephritis in order to guide health-care professionals on the optimal selection of an antimicrobial agent and its duration of therapy. The 2010 updated guidelines recommend four first-line therapies for uncomplicated cystitis: nitrofurantoin 100 mg orally twice daily for 5 days, trimethoprim-sulfamethoxazole 160 mg/800 mg orally twice daily for 3 days in areas where resistance to *Escherichia coli* does not exceed 20%, fosfomycin 3 grams single oral dose, or pivmecillinam 400 mg orally twice daily for 5 days. Fluoroquinolones and *β*-lactams remain as second-line agents. In Lebanon, pivmecillinam is not available, and resistance of *Escherichia coli* to TMP-SMX is approximately 50% rendering it an unsuitable empirical treatment for uncomplicated cystitis.

Similar studies evaluating antibiotic prescribing practices for uncomplicated cystitis in the United States reveal a low adherence to the guidelines and an increase in the use of fluoroquinolones for this indication [6, 15]. Fluoroquinolones, mainly ciprofloxacin and levofloxacin, have a broad spectrum of activity against uropathgones, and have been shown to be highly efficacious in 3-day regimens. They also possess moderate activity against enterococcus, a Gram-positive organism often complicating UTIs [16]. Moreover, fluoroquinolones possess favourable pharmacokinetics including high concentrations in the urinary tract, good oral bioavailability, and good renal excretion. However, these drugs have been associated with MRSA infections and an increase in the resistance of difficult-to-treat Gram-negative bacilli such as *Pseudomonas aeruginosa* [7]. Several case studies have shown a significant association between prior levofloxacin or ciprofloxacin use and the emergence of a subsequent MRSA infection in hospitalised patients [17].

Patients with uncomplicated cystitis have a minimal risk of progression to severe disease and generalized infection. Also, uncomplicated cystitis is one of the most common indications for antibiotic use. Consequently, episodes of selection of drug-resistant organisms repeated many times may cumulatively magnify the impact of collateral damage. For these reasons, it is of significant importance to consider the risk of collateral damage when using fluoroquinolones.

Our study demonstrates that, in Lebanon, physicians have not fully modified their prescribing practices to be in conformance with the guidelines. According to our study, the most frequently prescribed drug class was fluoroquinolones (39%), with ciprofloxacin being the most frequently prescribed fluoroquinolone (49%). Nitrofurantoin was the most commonly prescribed drug (26%), and a total of only 19 patients (5%) were prescribed fosfomycin. Overall appropriate regimens were seen in only 21% of the patients. The choice of the appropriate antibiotic was negatively affected by the number of UTI attacks per year. Interestingly, inappropriate doses and duration of therapy were more significantly observed with fluoroquinolones.

Fluoroquinolones have also been increasingly associated with adverse effects. The Food and Drug Administration (FDA) conducted a review of placebo-controlled clinical trials and a search of the FDA Adverse Event Reporting System database from November 1997 to May 2015, and they identified 178 patients in the United States who developed a disability or a potentially irreversible side effect after the use of a fluoroquinolone to treat acute bacterial sinusitis (ABS), acute bacterial exacerbation of chronic bronchitis (ABECB), or uncomplicated UTI. As a result, the FDA issued a warning regarding the use of fluoroquinolones for patient safety concerns. This warning mandated that drug labels and medication guides be updated. Now, the Indications and Usage section contains new limitation-of-use statements to reserve fluoroquinolones for patients who do not have alternative treatment options for ABS, ABECB, and uncomplicated UTI. The Warnings and Precautions sections of the labels include the serious risk of disabling and potentially irreversible adverse reactions such as tendinitis and tendon rupture, muscle pain, muscle weakness, joint pain, joint swelling, peripheral neuropathy, and central nervous system effects [18].

We also found that fosfomycin was not frequently prescribed in spite of the guideline's recommendations listing it as a first-line antibiotic. Fosfomycin is advantageously given as a single oral dose, increasing patient compliance, and the resistance of *Escherichia coli* to fosfomycin is very low in Lebanon [19]. Additionally, resistance to fosfomycin observed in clinical studies appears to be considerably lower than the resistance seen in in-vitro data [20]. In urinary tract infections in particular, the development of resistance is low because of the increased drug concentration in the acidic urinary pH. This is possibly due to the low adherence of fosfomycin-resistant mutants to epithelial cells. The lower resistance pattern to fosfomycin seen in clinical trials can also be attributed to the reduced growth and virulence of the mutant strains compared to the parent strains. This was shown to be mainly true not only for *Escherichia coli,* but also for *Klebsiella pneumoniae* and *Proteus mirabilis*.

Grigoryan and Zoorab examined data from two private family medicine faculty clinics from 2011 to 2014 [21]. They assessed the choice of antibiotic and the duration of treatment administered for acute uncomplicated UTI. Fluoroquinolones were the most commonly prescribed antibiotics (51.6% of visits), followed by nitrofurantoin (33.5%), TMP/SMX (12%), and other antibiotics (3.2%). Regarding the duration of treatment, 71% of the prescriptions for fluoroquinolones, 82% of the prescriptions for TMP/SMX, and 76% of the prescriptions for nitrofurantoin were given for a duration that exceeded the guidelines' recommendations [21].

Another cross-sectional study, involving 61 patients, gathered data from July 2011 to June 2012 in a university-based internal medicine clinic [3]. According to this study, the overall concordance for the entire regimen with the IDSA 2010 updated guidelines was 34%. TMP/SMX was the most frequently prescribed antibiotic (45.3%), followed by ciprofloxacin (28.3%) and nitrofurantoin (24.5%). Interestingly, more than half of the patients prescribed TMP/SMX received a regimen in complete concordance with the guidelines, as opposed to none of the patients prescribed ciprofloxacin [3].

Our findings are in line with previous studies showing low adherence to the IDSA 2010 guidelines for the treatment of uncomplicated cystitis. Moreover, studies published before the update also showed low adherence to the 1999 version of the IDSA guidelines, suggesting that the updated guidelines may not have significantly altered prescribing practices [7].

Also, in Lebanon, it should be noted that fluoroquinolones are relatively more expensive than other available treatment options such as nitrofurantoin and fosfomycin. The increase in the use of fluoroquinolones not only confers a risk of increased cost and collateral damage, but also poses an increased risk of serious adverse effects.

The low adherence to the IDSA guidelines could be due to a lack of awareness to the recommendations, physicians' familiarity and preference for certain antibiotics based on their clinical experience, and concern for infectious complications. It may also be due in part to the difficulty of keeping up with new recommendations for many different diseases.

To our knowledge, this is the first study to evaluate antibiotic-prescribing practices and adherence to IDSA guidelines for the treatment of uncomplicated UTI in Lebanon. Nevertheless, the study has several limitations. First, it is observational in nature so the cause-effect relationship between elements could not be ensured. Second, convenience samples of only fifteen Lebanese community pharmacies were included in the study. However, patients were recruited from different geographical areas and for a one-year period which could dilute this limitation and make the sample representative. Finally, there was no follow-up of patients after filling-in their prescriptions which could have provided more information about the antibiotic's efficacy and safety.

## 5. Conclusions

This study demonstrates a high prevalence of inappropriate use of antibiotics for the treatment of outpatient uncomplicated urinary tract infections in Lebanon. This is mainly attributable to inappropriate indication, dose, and/or duration of therapy with fluoroquinolones. In an era of increasing bacterial resistance and dwindling antimicrobial choices for Gram-negative infections, interventions that improve physicians' prescribing practices through education on appropriate therapy for uncomplicated UTIs are needed.

## Figures and Tables

**Table 1:** Baseline demographics.

Demographics	Number of patients	Percentage
*Age*		
18–30	117	31
30–40	114	30
40–50	84	22
50–60	39	10
>60	22	6
*Number of UTI per year*		
1	194	52
2	113	30
≥3	69	18
*Prescribed antibiotic*		
Fluoroquinolones	146	39
Nitrofurantoin	98	26
TMP-SMX	52	14
Cephalosporin	40	11
Fosfomycin	19	5
Amoxicillin/clavulanate	12	3
Other *β*-lactams	5	1
Other antibiotics	4	1
*Allergy*		
Penicillin	28	7
TMP-SMX	4	1
Nitrofurantoin	1	0
Fluoroquinolones	1	0
Tetracyclines	1	0
No allergy	341	91
*Nonpharmacological treatment*		
Yes	276	73
No	100	27
*Culture obtained*		
Yes	97	26
No	279	74

**Table 2:** Appropriateness of treatment.

	Appropriate	Nonappropriate
Regimen	80 (21%)	296 (79%)
Drug	131 (35%)	245 (65%)
Dose	276 (73%)	100 (27%)
Duration	219 (58%)	157 (42%)

**Table 3:** Bivariate analysis.

	Appropriate antibiotic		Appropriate dose		Appropriate duration		Appropriate overall regimen	
*Age*								
<50 years	112 (35.6%)	*p*=0.508	232 (73.7%)	*p*=0.806	183 (58.1%)	*p*=0.894	66 (21.0%)	*p*=0.727
>50 years	19 (31.1%)		44 (72.1%)		36 (59%)		14 (23.0%)	
*Frequency of attacks*								
<3/year	115 (37.5%)	*p*=0.025	226 (73.6%)	*p*=0.845	180 (58.6%)	*p*=0.748	72 (23.5%)	*p*=0.30
≥3/year	16 (23.2%)		50 (72.5%)		39 (56.5%)		8 (11.6%)	
*Choice of medication*								
Nitrofurantoin	N/A		76 (77.6%)	*p* < 0.001	58 (59.2%)	*p*=0.014	N/A	
Fosfomycin	N/A		19 (100%)		15 (78.9%)		N/A	
Fluoroquinolones	N/A		80 (54.8%)		72 (49.3%)		N/A	
